# Euthanasia and the rehabilitation of wildlife casualties in Finland: decision-making varies depending on the background education of the caregivers

**DOI:** 10.3389/fvets.2023.1207930

**Published:** 2023-07-26

**Authors:** Kati White, Laura Hänninen, Anna Valros

**Affiliations:** ^1^Research Centre for Animal Welfare, Faculty of Veterinary Medicine, Helsinki University, Helsinki, Finland; ^2^SEY, Animal Welfare Finland, Helsinki, Finland

**Keywords:** wildlife, rehabilitation, euthanasia, ethics, decision-making

## Abstract

Caring for wildlife casualties is a common aspect of animal-protection work. The range of care options of wildlife in Finland vary from professional zoos to voluntary members of the public. There are complex ethical concerns to be considered in deciding whether an injured animal should be treated or euthanized. Differing opinions and poor communication may lead to unnecessary conflicts among caregivers. We investigated opinions behind the decision-making of caregivers related to wildlife casualties using a web-based questionnaire. We asked the respondents to rate their level of agreement with 27 statements on a seven-point Likert scale. Seventy-eight respondents were included in our analysis. Animal-related education was classified as veterinarian (*n* = 14), other (*n* = 18), and none (*n* = 49). The median (IQR) levels of age and work experience were 43 (17) and 5 (9) years, respectively, regardless of educational level. The groups were tested for differences level of agreement with the statements in Kruskall-Wallis tests (with Bonferroni-corrected pair-wise tests). Overall, the strongest disagreement was with statements proposing euthanasia on the grounds that the species was common [1 (2)], the treatment would be costly [1 (4)] or long-term [1 (4)], or there was no end-of-life-solution immediately available [1 (2)]. The highest agreement was with the statement advocating not euthanizing the animal if it could easily be returned to its natural habitat [7 (0)]. The respondents differed in their perceptions depending on their animal-related education. The cost and length of treatment, the prevalence of the species, and a known end-of-life solution influenced the euthanasia-related decisions of veterinarians more than of respondents in the other educational groups. Those with no animal-related education expressed the least willingness to euthanize an injured wild animal, even if it would be partly dependent on humans for the rest of its life or even if the treatment would be very stressful. We concluded that attitudes and practices related to euthanasia differ depending on the respondents’ education, and that more discussion is needed on the ethical aspects behind the decision-making. This would help to increase mutual understanding among caregivers and facilitate the development of uniform standards that would potentially benefit animal welfare.

## 1. Introduction

Wildlife rescue is part of animal welfare work in many countries around the world (1). Some countries have specific requirements concerning the rehabilitation of wildlife (2, 3). In Finland however, there are no consistent standards, nor are permits required. Although there are some contexts, such as zoos, in which wildlife casualties are treated professionally, this is generally based on voluntary work carried out by NGOs and members of the public. Members of the veterinary profession also have an important role in treating wildlife in Finland and in many other countries, being the only people with the right to prescribe medication (4–6). It seems that practices, expectations, and quality of treatment vary widely among caregivers in every aspect of the process of caring for wildlife casualties. This, together with poor communication, may lead to discrepancies, conflicts, and lower-level cooperation between veterinarians and volunteers to the best of our knowledge (7). Thus far, however, there have been no studies on how stakeholders with different educational backgrounds perceive factors affecting wildlife care in Finland.

Among the factors affecting wildlife care, there are complex ethical concerns to be carefully considered when decisions are made concerning whether to treat or euthanize a wild animal, or whether humans should intervene at all (8). Although there is a strong moral, and in many countries a legal obligation to help animals for which humans are responsible (1, 9), the threshold for intervention in the case of wild animals may vary significantly (10). Opinions on what is acceptable differ among stakeholders (8), and conflicts may arise due to disagreement on the subject of euthanasia, for example (7). Some people may think that the prime aim must always be to return an animal successfully to the wild (8), and that euthanasia must be considered if at any stage of the rehabilitation process it seems that the animal is unlikely be safely returned, or if the treatment is causing excessive distress (9, 11, 12). Others, on the other hand, argue that the welfare of wild animals can be maintained on an acceptable level in captivity, although this strongly depends on individual aspects such as the species in question and the conditions of captivity (13). It should also be considered whether the rehabilitation process should be started if there is the slightest chance that the animal will survive, and what is an acceptable level of stress and pain to be suffered during the rehabilitation process. It has been suggested that the welfare of the individual patient should be the priority in the treatment of wild animals (14). In any case, assessments and decisions should be made as soon as possible when a wildlife casualty is presented, not only to prevent its suffering but also to ensure staff safety (8).

There is thus a need to develop uniform standards of treatment, which in turn would require more information about why wildlife require care or are euthanized, what factors affect the decision-making, and how decisions might differ among people dealing with wildlife casualties. The aim of this study was to assess opinions related to euthanasia among wildlife casualty caregivers in Finland, and to assess if opinions differ between people with different educational backgrounds. This study is part of a more extensive project investigating the practices and perspectives of persons administering treatment to wildlife.

## 2. Materials and methods

In spring 2020 we posted a web-based, anonymous questionnaire (Qualtrics^xm^, Seattle, USA) aimed at Finnish Facebook groups for veterinarians and volunteers caring for wildlife. The questionnaire was open for approximately 1 month. We were also in direct e-mail contact with professional rehabilitators in zoos and volunteer caregivers practicing within the biggest Finnish animal welfare organization, Animal Welfare Finland SEY to reach more participants.

### 2.1. Questionnaire

This questionnaire was part of a larger one comprising 12 sections with questions concerning euthanasia and wildlife and taking wild animals in for treatment and rehabilitation. Questions were mainly closed, and opinion-related questions applied a Likert scale (1-7). In addition, the questionnaire included a few open text questions. In the absence of a readily available questionnaire, we formulated the items based on our aims. The larger questionnaire covered areas such as species the respondent is taking care of; reasons for which animals are brought to them and factors affecting whether animals are taken in for treatment; how have the respondents gained their knowledge on caregiving; numbers of animals that are rehabilitated back to wild or other solutions taken; in which cases is veterinarian consulted; and what kind of treatments or medications do they provide themselves. Before launching the questionnaire, we sent the questionnaire for feedback to four non-veterinarians and four veterinarians taking care of wildlife casualties to make sure that the content was appropriate, and the questionnaire was slightly modified accordingly.

Here we report on the responses to questions concerning the perceived reasoning behind decisions to euthanize wild animals. This included 27 statements in 3 sections out of the 12 sections of the whole questionnaire. We asked the respondents to think about a typical individual wildlife patient for whom euthanasia was being considered, and to score their agreement on claims regarding that situation on a seven-point Likert scale, on which 1 corresponded to complete disagreement and 7 to complete agreement. The larger questionnaire also included a set of items on background factors. Of these, we analyzed for differences between people with differing animal-related education. To make sure differences are not due to other demographic factors, we also included information on gender, age (birth year), and experience of treating wild animals in numbers of years. We included all respondents who had answered the three sections of questions analyzed here. All respondents had recent history of taking care of wildlife casualties.

Responses were anonymous. We followed the guidelines of the Finnish National Board of Research Integrity (TENK)^1^, according to which no ethical review was required.

### 2.2. Statistical analysis

Before conducting the statistical analyses we classified the respondent’s animal-related education as veterinarian, other (such as trained caretaker, biologist, or veterinary nurse), and none.

Because the data did not follow a normal distribution, we used Kruskal-Wallis tests to assess the differences between the educational groups in their ratings of the statements, their age and work experience, and χ^2^-tests to identify possible gender differences. Pair-wise comparisons were Bonferroni-corrected. We used IBM SPSS Statistics version 27 (IBM Inc. Chicago, IL) for analyzing the data. Significance was declared at *p* ≤ 0.05.

## 3. Results

Of the 119 respondents, 78 had answered enough of the questions to be included in the analysis. Of these, 14 were veterinarians, 18 had other animal-related education, and 49 had no animal-related education. The respondents’ median (interquartile range) age and work experience were 43 (17) years and 5 (9) years, respectively, with no differences between the educational groups (*p* > 0.1 for all). Regarding gender, 91 percent of the respondents identified as female, 7.7 percent as male, and 1.3 percent as other, with no differences between the educational groups.

**Figure 1:**
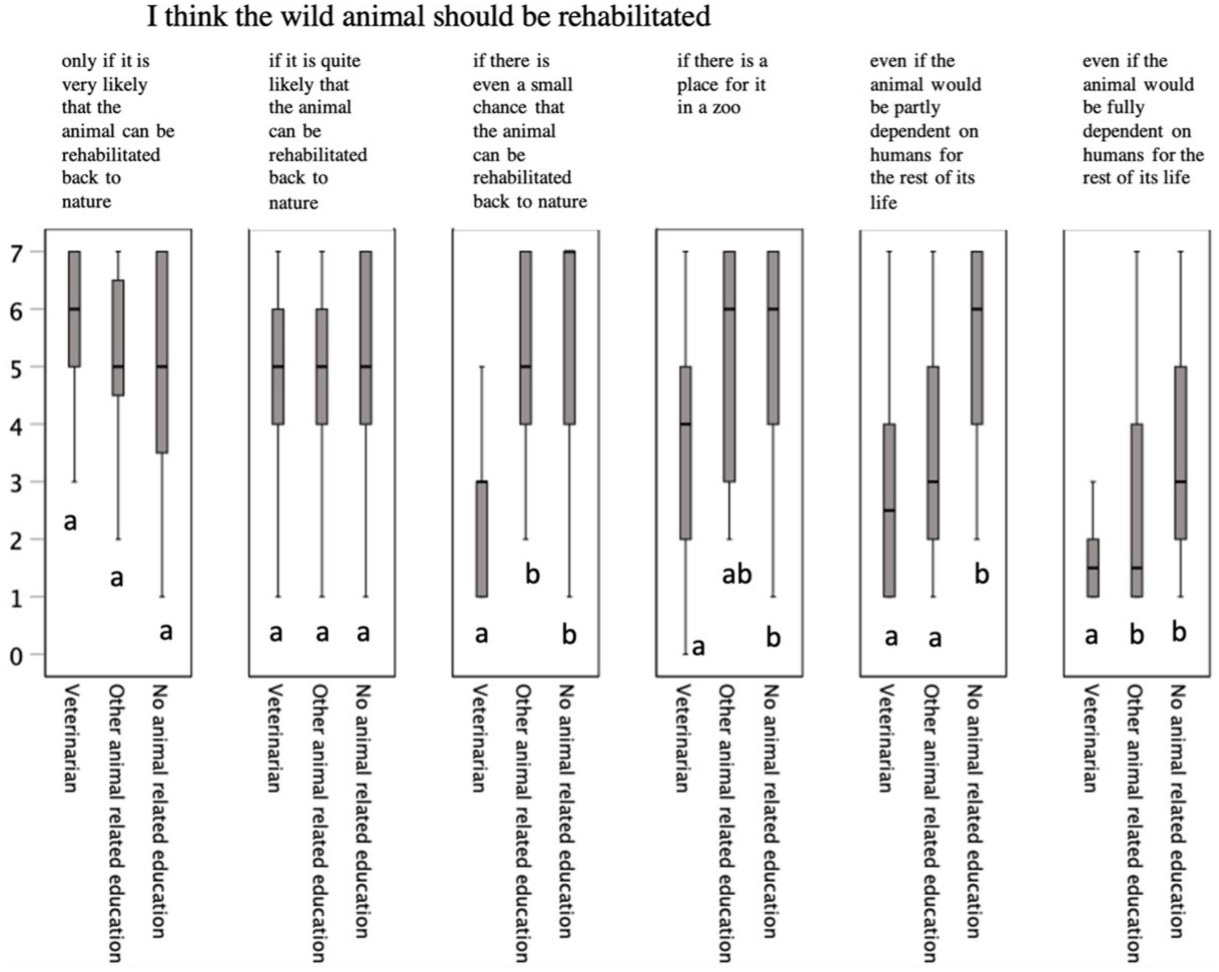
Median (IQR, interquartile range) differences in animal-rehabilitation-related statements among persons with different educational backgrounds treating injured wildlife (*n* = 78, Likert-scale in which 1 represents complete disagreement, 7 represents complete agreement). Bars within the graph lacking a common letter differ statistically significantly.

Table 1 shows overall descriptive results for all the statements. Overall, the highest levels of disagreement among the respondents related to the statements proposing euthanasia because the species was common [median: 1 (interquartile range: 2)], because of the expected high treatment price [1 (4)] or long treatment duration 1 (4), or if there was no end-of-life-solution immediately available [1 (2)]. On the other hand, the respondents expressed the highest level of agreement with the statement that proposed not euthanizing the animal if it could easily be returned to the wild [7 (0)].

**Table 1:** Overall median (interquartile range, IQR) agreement among persons treating injured wildlife on euthanasia-related questions (*n* = 78, Likert-scale, in which 1 represents complete disagreement and 7 complete agreement).

Statements	Median agreement (IQR)
If the costs of the treatment are starting to become high, I end up euthanizing the animal.	1 (4)
I end up with euthanasia if the animal’s ailment requires more than half a year of treatment and the animal is not of a species that hibernates.	1 (4)
I end up with euthanasia if the animal’s ailment requires more than half a year of treatment during which it would hibernate for part of the time.	1 (4)
If there is no known end-of-life solution for the animal at the time it is brought to me, in my opinion it should be euthanized immediately regardless of the species and of its condition.	1 (2)
If there is no known end-of-life solution for the animal at the time it is brought to me, it should be euthanized immediately if the species in question is common.	1 (2)
Whether or not the animal will survive in the wild after treatment does not have any impact on my decision related to euthanasia.	2 (4)
I think a wild animal should be rehabilitated even if will be fully dependent on humans for the rest of its life.	2 (5)
I end up with euthanasia if I believe that the treatment will cause the animal a lot of stress.	3 (2)
I think a wild animal should be rehabilitated even if it will be partly dependent on humans for the rest of its life.	3 (5)
I do not as easily euthanize endangered species, and I try to treat them longer than I treat less endangered species.	4 (5)
If there is no known end-of-life solution for the animal at the time it is brought to me I will start the treatment, but only if it is unlikely to stress the animal very much.	4 (4)
If there is no known end-of-life solution for the animal at the time it is brought to me I could start treating it whatever its species and condition, but any end-of-life solution should be known within 2 days at the most.	5 (6)
If there is no known end-of-life solution for the animal at the time it is brought to me I would start treatment only if it was unlikely to cause a lot of pain and suffering.	5 (4)
I think a wild animal should be rehabilitated only if it is very likely to return to the wild.	5 (3)
I think a wild animal should be rehabilitated if it is quite likely to be returned to the wild.	5 (3)
I think a wild animal should be rehabilitated even if there is only a small chance that it will be returned to the wild	5 (4)
I think a wild animal should be rehabilitated if there is a place for it in a zoo.	5 (4)
I end up with euthanasia if I think that the treatment will cause the animal a lot of pain.	6 (3)
If there is no known end-of-life solution for the animal at the time it is brought to me, whether or not it is of an endangered species, treatment can start while an end-of-life solution is being sought.	6 (3)
If there is no known end-of-life solution for the animal at the time it is brought to me, and it is of an endangered species, it can be treated indefinitely until an end-of-life solution is found.	6 (4)
It is unlikely that I would euthanize the animal if it could easily be returned to live in the wild.	7 (0)

We identified several differences in the respondents´ perceptions based on their animal-related education (see Table 2). The cost and length of the treatment, the prevalence of the treated species and a known end-of-life solution for the injured animal influenced the euthanasia decisions of veterinarians more than of those in the other educational groups. Respondents with no education related to animals would be the least willing to euthanize an injured wild animal, even if it would be partly dependent on humans for the rest of its life and the treatment would be very stressful.

**Table 2:** Median (IQR) differences in euthanasia-related statements among persons with different educational backgrounds treating injured wildlife (*n* = 78, Likert-scale on which 1 represents complete disagreement and 7 complete agreement); IQR: interquartile range; the numbers within a row lacking a common letter differ statistically significantly.

Statements	Median (IQR)			*p*	H (df = 2, *n* = 78)
	Veterinarian	Other animal-related education	No animal-related education		
**I do not as easily euthanize endangered species, but I try to treat them for longer than less endangered species.**	**5.50(1.50) a**	**3.50(4.75) ab**	**3.00(4.00) b**	**0.019**	**7.88**
**If the costs of the treatment are starting to become high, I end up euthanizing the animal.**	**4.00(3.00) a**	**1.00(2.00) b**	**1.00(2.00) b**	**<0.001**	**20.23**
**I end up with euthanasia if I believe that the treatment will cause the animal a lot of stress.**	**5.00(2.00) a**	**5.00(2.00) a**	**3.00(3.00) b**	**<0.001**	**19.15**
Whether or not the animal will survive in the wild after treatment does not have any impact on my decision to euthanize.	2.00(1.00)	2.00(5.00)	2.00(3.00)	ns	1.26
I end up with euthanasia if I believe that the treatment will cause the animal a lot of pain.	6.50(1.00)	6.00(2.00)	6.00(3.00)	ns	3.34
It is unlikely that I will euthanize the animal if it can easily be returned to live in the wild.	7.00(1.00)	7.00(0.00)	7.00(0.00)	ns	0.23
**I end up with euthanasia if the animal’s ailment requires more than half a year of treatment, and if it is not of a species that hibernates.**	**6.50(2.00) a**	**2.00(3.00) b**	**1.00(2.00) b**	**<0.001**	**23.72**
**I end up with euthanasia if the animal’s ailment requires more than half a year of treatment during which it would hibernate for part of the time.**	**4.00(3.00) a**	**1.00(2.00) b**	**1.00(1.00) b**	**<0.001**	**20.84**
**If there is no known end-of-life solution for the animal when it is brought to me, it should be euthanized immediately whatever its species and condition.**	**3.00(2.00) a**	**1.00(2.00) ab**	**1.00(0.00) b**	**0.003**	**11.9**
**If there is no known end-of-life solution (setting it free in the wild, a place in an enclosure for wild animals) for the animal when it is brought to me, I will start treatment regardless of its species and condition, but any end-of-life solution should be known within 2 days at the most.**	**5.00(3.00) a**	**2.00(3.00) ab**	**2.00(4.00) b**	**0.021**	**7.68**
**If there is no known end-of-life solution (setting it free in the wild, a place in an enclosure for wild animals) for the animal when it is brought to me, regardless of the species and whether it is endangered or not, treatment can begin until an end-of-life solution is found.**	**3.5(4) a**	**7.00(1.00) b**	**7.00(1.00) b**	**<0.001**	**16.57**
**If there is no known end-of-life solution (setting it free in the wild, a place in an enclosure for wild animals) for the animal when it is brought to me, if it is of an endangered species, it could be treated indefinitely until an end-of-life solution is found.**	**4.00(3.00) a**	**5.00(4.00) ab**	**7.00(2.00) b**	**0.005**	**10.67**
**If there is no known end-of-life solution (setting it free in the wild, a place in an enclosure for wild animals) for the animal when it is brought to me, it should be euthanized immediately if it is of a common species.**	**3.00(4.25) a**	**1.00(1.00) b**	**1.00(0.00) b**	**<0.001**	**15.52**
If there is no known end-of-life solution (setting it free in the wild, a place in an enclosure for wild animals) for the animal when it is brought to me, treatment can begin only if it is unlikely to cause the animal a lot of stress.	5.00(1.00)	3.00(3.00)	4.00(4.00)	ns	3.68
If there is no known end-of-life solution (setting it free in the wild, a place in an enclosure for wild animals) for the animal when it is brought to me, treatment can begin only if it is unlikely to cause it a lot of pain and suffering.	5.50(2.00)	5.00(3.00)	5.50(3.00)	ns	0.64

Bolded rows indicate comparisons where overall *p* < 0.05, ns: *p* > 0.05.

Although all educational groups were ready to return the animal to the wild if it was most likely or very likely to survive, there were differences between them in opinions related to other rehabilitation situations (*p* < 0.05 for all, Figure 1).

## 4. Discussion

Views related to animal euthanasia decisions varied among caregivers of wildlife in Finland, depending on whether they had any animal-related education or not. The low overall median agreements with the statements regarding euthanizing animals if they could be easily returned to live in the wild; because the species was common; or because of expected high treatment costs or extended treatment time, all indicate respect for the life of wildlife animals. This is also supported by the low agreement with the statement that the lack of an immediate end-of-life-solution motivates euthanasia.

Among other things, we found among respondents with no animal-related education that euthanasia decisions were not strongly influenced by whether or not the treatment would stress an injured wild animal, or by the fact that the animal would be dependent on people for the rest of its life. Education may help caregivers to identify species-specific needs and signs of pain and stress among injured wildlife. We suggest that the deeper understanding of animal suffering gained from education may also sensitize some individuals to the suffering of animals, which could lead to lower threshold for euthanasia decisions. As Batchelor and McKeegan (15) report, for example, veterinarians in the UK suggest that prolonged care of a sick animal causes more stress than euthanizing a healthy animal. Animal-related training, including veterinary training, does not necessarily ensure that the people concerned fully understand the effect of rehabilitation on wildlife casualties, however. Hearing (7) reported that Australian veterinarians did not think their formal training gave them enough knowledge about wildlife rehabilitation, and that many complaints are received from wildlife rehabilitation groups claiming that veterinarians prolong the lives of animals that should be euthanized if the code of practice for Injured, Sick and Orphaned Protected Fauna (2) were to be followed.

We also found that, when making their treatment decisions and considering its length, veterinarians placed more value than other respondents on whether or not the animal was of an endangered species. Resources are often limited, requiring choices to be made about which animals to treat. Saving an animal belonging to an endangered species may have added value related to protecting the whole species. Moreover, some may question the decision to rehabilitate an animal of a species whose population is to be reduced (16).

Overall, veterinarians were more willing to euthanize an injured individual wild animal than respondents in the other educational groups, whereas those with no animal-related education were the least willing. We suggest that the ethical approach, whether it be utilitarian, deontological or other, may affect these attitudes. We cannot rule out the possibility that utilitarian-minded people may seek to join the veterinary profession in the first place: this is partly supported in our study, in which Finnish veterinary students typically have a utilitarian approach to ethical questions (17). Proponents of utilitarianism seek solutions that lead to the greatest good for the greatest number of stakeholders (10). Therefore, practical reasons such as the high cost, the expected length, and the potential success of rehabilitation, as well as the status of the species in question, may have a bigger impact on the decision-making than if the approach had been deontological, meaning that the overall consequences matter less than individual rights (10). Non-veterinarian respondents treating wild animals in Finland may be more representative of the deontological approach than veterinarians. There has been no published research on this issue, however. More studies are needed to complement current knowledge on the rationale behind decisions, thereby enhancing understanding and easing conflicts among caregivers and improving animal welfare.

One obvious reason why veterinarians are more likely to euthanize injured wildlife than caregivers in other educational groups is that euthanasia is easier for them to deal with. They are trained to accept euthanasia as a valid choice of treatment (18), and it is sometimes used on animals for reasons of convenience (19, 20). Veterinarians are also used to making decisions related to euthanasia and balancing different interests, having to face these situations in their daily work (21, 22). For some, euthanasia is a relief among many other less controlled options such as serious complications, unintended harm, suffering and a non-peaceful death (23). Veterinarians participating in a Canadian research project reported that a “good death,” meaning ending animal suffering and everything going smoothly during the process, had positive effects on their own welfare (24). It may be that veterinarians in many countries, including Finland, generally have a more positive attitude toward euthanasia as a choice of treatment than other stakeholders. Moreover, Finland has no legal restrictions on its use in this context, and even healthy animals can be euthanized.

Veterinarians were the most strict in their view that injured animals should be relocated back to the wild, or euthanized, and they were reluctant to treat an animal if there was no end-of-life solution available within the following 2 days. Here, their views also differed from those of respondents with other than veterinary training, these formed a heterogeneous group consisting of volunteers and zoo and Animal Clinic staff. However, veterinarian approach was in line with the recommendation that assessments and decisions related to wildlife casualties should be made as soon as possible to prevent suffering, but also for reasons of staff safety, for example (8). It has been suggested that euthanasia should be considered at any stage of rehabilitation if it becomes unlikely that the animal can be safely returned to the wild, if it would be permanently disabled, or to prevent further suffering (11) or if the treatment would cause excessive distress (12). Rates of returning wildlife casualties have been 40 and 46 per cent in the UK and the Czech Republic, respectively (12, 16). Exceptions to this goal could be considered if the quality of life in a captive or semi-captive environment could be assured (12). Given that a large proportion of animals cannot be released, it is important to determine with some accuracy which individuals should be treated to avoid unnecessary suffering, and what would be the most efficient use of resources for wildlife rehabilitation (12, 16). In line with these aspirations, recently adopted Finish animal-welfare law states that a wild animal casualty must be helped, but if keeping it alive amounts to cruelty, it has to be euthanized (9).

The number of respondents was rather low, especially among veterinarians, making it difficult to generalize the results. However, we cannot assess the actual response rate, as there is no information available on the total number of people, including veterinarians, taking care of wildlife in Finland. According to our best knowledge, however, the total number is not very high, and we did our best to reach all people who work on wild animals either professionally or voluntarily. Even though we do not see any reason to suspect a bias due to the way of distribution of the questionnaire, we regrettably have no way of controlling for response bias, which might have been affected by the caregivers’ own activity and interest in the topic. This study indicates that to reduce possible conflicts and to improve animal welfare there is a need for more studies on the rationale behind decision making, as well as on the ethical justifications for decisions, among wildlife caregivers and stakeholders.

## 5. Conclusion

We have shown here that there are differences in attitudes related to decision-making on the question of euthanizing wildlife casualties, and that the differences relate to the background education of the respondents. More discussion is needed on the ethical aspects behind the decision-making to increase understanding among caregivers, and thereby to develop more uniform standards that could potentially benefit animal welfare.

## Data availability statement

The raw data supporting the conclusions of this article will be made available by the authors, without undue reservation.

## Ethics statement

Ethical review and approval was not required for the study on human participants in accordance with the local legislation and institutional requirements. The patients/participants provided their written informed consent to participate in this study.

## Author contributions

KW drafted and tested the questionnaire, interpreted the results, and wrote the first draft of the manuscript. LH analyzed the data, helped with drafting the manuscript and supervised. AV participated in the drafting of the questionnaire, supervised, and helped draft the manuscript. All authors contributed to the article and approved the submitted version.

## Conflict of interest

The authors declare that the research was conducted in the absence of any commercial or financial relationships that could be construed as a potential conflict of interest.

## Publisher’s note

All claims expressed in this article are solely those of the authors and do not necessarily represent those of their affiliated organizations, or those of the publisher, the editors and the reviewers. Any product that may be evaluated in this article, or claim that may be made by its manufacturer, is not guaranteed or endorsed by the publisher.

## Footnotes

^1^http://www.tenk.fi/en/tenk-guidelines

## References

[1] Kirkwood JK. Introduction: wildlife casualties and the veterinary surgeon In: Mullineaux E Best D Cooper JE, editors. BSAVA manual of wildlife casualties. Gloucester, MA, USA: BSAVA Publications (2003). 1–5.

[2] New South Wales Office of Environment and Heritage. Code of practice for injured, sick and orphaned protected Fauna. (2011). Available at: https://www.environment.nsw.gov.au/-/media/OEH/Corporate-Site/Documents/Licences-and-permits/code-practice-injured-protected-fauna-110004.pdf (Accessed January 20, 2023).

[3] Hammarberg K Hillarp J-Å. Frågor och svar om sjukt eller skadat vilt In: Så tar du hand om skadat vilt. Malmö, Sweden: Djurdoktorn (2019). 7.

[4] Finnish Ministry of Agriculture and Forestry. Act on Access to and Pursuit of the Profession of Veterinary Surgeon 29/2000. (2000). Available at: https://www.finlex.fi/fi/laki/ajantasa/2000/20000029 (Accessed January 20, 2023).

[5] U.S. Food and Drug Administration. FDA regulation on animal drug. (2022). Available at: https://www.fda.gov/animal-veterinary/resources-you/fda-regulation-animal-drugs#dispensing (Accessed January 20, 2023).

[6] UK Government, Veterinary Medicines Directorate. Collection Veterinary medicines guidance. (2015). Available at: https://www.gov.uk/government/collections/veterinary-medicines-guidance-notes-vmgns (Accessed January 20, 2023).

[7] Haering R Wilson V Zhuo A Stathis P. A survey of veterinary professionals about their interactions with free-living native animals and the volunteer wildlife rehabilitation sector in New South Wales, Australia. Australian Zool. (2021) 41:254–82. doi: 10.7882/AZ.2020.045

[8] Meredith A. Wildlife triage and decision making for the general practitioner, world small animal veterinary association world congress proceedings. (2008). Available at: https://www.vin.com/apputil/content/defaultadv1.aspx?id=3866644&pid=11268& (Accessed January 20, 2023).

[9] Finnish Government. HE 186/2022 Hallituksen esitys eduskunnalle laiksi eläinten hyvinvoinnista ja siihen liittyviksi laeiksi (Unofficial translation: Governments proposal to the Parliament on law for animal welfare and related laws). (2022). Available at: https://www.eduskunta.fi/FI/vaski/HallituksenEsitys/Sivut/HE_186+2022.aspx (Accessed January 20, 2023).

[10] Mullan S. Fawcett A. Veterinary ethics: navigating tough cases. Sheffield, UK: 5M Publishing Ltd (2017). p 453–482.

[11] Mullineaux E Best D. Basic principles of treating wildlife casualties In: Mullineaux E Best D Cooper JE, editors. BSAVA manual of wildlife casualties. Gloucester, MA, USA: BSAVA Publications (2003). 6–28.

[12] Grogan A Kelly A. A review of RSPCA research into wildlife rehabilitation. Vet Rec. (2013) 172:211. doi: 10.2236/vr.101139, PMID: 23436601

[13] Browning H Veit W. Freedom and animal welfare. Animals. (2021) 11:1148. doi: 10.3390/ani11041148, PMID: 33920520 PMC8073385

[14] Mullineaux E. Veterinary treatment and rehabilitation of indigenous wildlife. J Small Anim Pract. (2014) 55:293–300. doi: 10.1111/jsap.12213, PMID: 24725160

[15] Batchelor C McKeegan D. Survey of the frequency and perceived stressfulness of ethical dilemmas encountered in UK veterinary practice. Vet Rec. (2012) 170:19. doi: 10.1136/vr.100262, PMID: 22084032

[16] Lukesova G Voslarova E Vecerek V. Mammals at rescue centres in the Czech Republic: trends in intake and outcome, causes of admission, length of stay and release rate. J Nat Conserv. (2022) 67:126156. doi: 10.1016/j.jnc.2022.126156

[17] Valros A Hänninen L. Animal ethical views and perception of animal pain in veterinary students. Animals. (2018) 8:220. doi: 10.3390/ani8120220, PMID: 30477084 PMC6315997

[18] Knesl O Hart B Fine A Cooper L Patterson-Kane E Houlihan KE. Veterinarians and humane endings: when is it the right time to euthanize a companion animal? Front Vet Sci. (2017) 4:45. doi: 10.3389/fvets.2017.00045, PMID: 28470002 PMC5395644

[19] Rollin B. Euthanasia, moral stress, and chronic illness in veterinary medicine. Vet Clin N Am Small Anim Pract. (2011) 41:651–9. doi: 10.1016/j.cvsm.2011.03.005, PMID: 21601753

[20] Littlewood K Beausoleil N Stafford K Stephens C Collins T Quain A. How decision-making about euthanasia for animals is taught to Australasian veterinary students. Aust Vet J. (2021) 99:334–43. doi: 10.1111/avj.13077, PMID: 34002368

[21] Tannenbaum J. Veterinary medical ethics: a focus of conflicting interests. J Soc Issues. (1993) 49:143–56. doi: 10.1111/j.1540-4560.1993.tb00914.x

[22] Kipperman B Morris P Rollin B. Ethical dilemmas encountered by small animal veterinarians: characterization, responses, consequences and beliefs regarding euthanasia. Vet Rec. (2018) 182:548. doi: 10.1136/vr.104619, PMID: 29445010

[23] Quain A. The gift: ethically indicated euthanasia in companion animal practice. Vet Sci. (2021) 8:141. doi: 10.3390/vetsci8080141, PMID: 34437463 PMC8402858

[24] Matte A Khosa D Coe J Meehan C. Impacts of the process and decision-making around companion animal euthanasia on veterinary wellbeing. Vet Rec. (2019) 185:480. doi: 10.1136/vr.105540, PMID: 31409747

